# Pontine Myopericytoma: Case Report and Literature Review

**DOI:** 10.3389/fonc.2022.903655

**Published:** 2022-05-16

**Authors:** Maoyuan Guo, Xue Chen, Gaofeng Zhang, Yongpeng Wang

**Affiliations:** Department of Radiology, The Second Affiliated Hospital of Zun Yi Medical University, Zunyi, China

**Keywords:** myopericytoma, pons, intracranial tumor, MRI, case report

## Abstract

Myopericytoma (MPC) is a benign soft tissue tumor that develops from perivascular myoid cells and is part of the perivascular tumor group. MPC most commonly occurs in the subcutaneous soft tissues of the extremities, while intracranial MPC is remarkably rare. Herein, we report the case of a 45-year-old woman with myopericytoma who had a 2-week history of recurrent dizziness. Magnetic resonance imaging (MRI) revealed an irregular mass in the pons, with nodular enhancement of the mass on contrast-enhanced scans. The mass was considered a vascular lesion and was highly suspected to be a hemangioblastoma, prompting surgical intervention for the patient. The postoperative pathological report corrected the initial diagnosis, hemangioblastoma, to MPC. Intracranial MPC is extremely rare and there are no detailed imaging sources for this condition; furthermore, MPC occurrence in the pons has not been reported previously. This report presents the etiological characteristics intracranial MPC as visualized through MRI data alongside a comparative discussion on other reported diagnoses that resemble MPC. The case findings will provide a more widespread understanding for radiologists regarding the differential diagnosis of intracranial blood-rich supply lesions.

## Case Presentation

**Figure 1:**
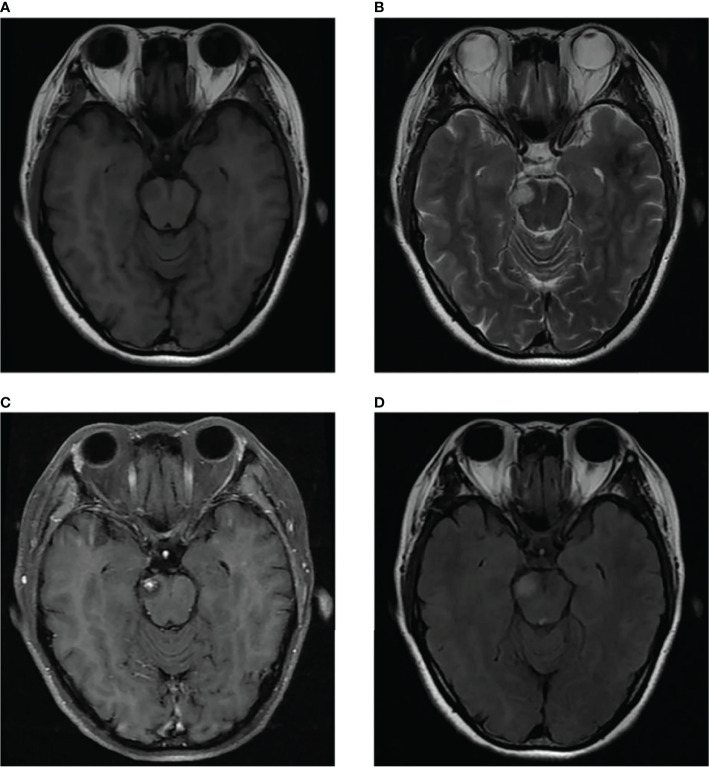
MRI scans showed a well-defined irregular mass in the pons **(B)**. The mass showed slight hyperintensity on T2-weighted MR images and FLAIR images **(B, D)** and comparatively lower intensity on T1-weighted MR images **(A)**. Contrast-enhanced MR image showed prominent heterogeneous enhancement of the lesion, presenting central enhancement **(C)**.

**Figure 2:**
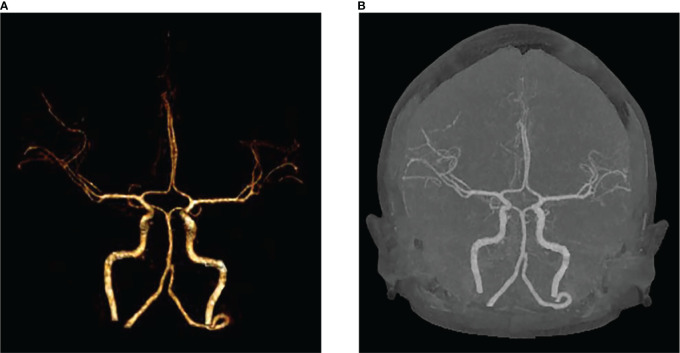
No significant abnormalities in the intracranial arteries on VRT **(A)** and MIP **(B)**.

**Figure 3:**
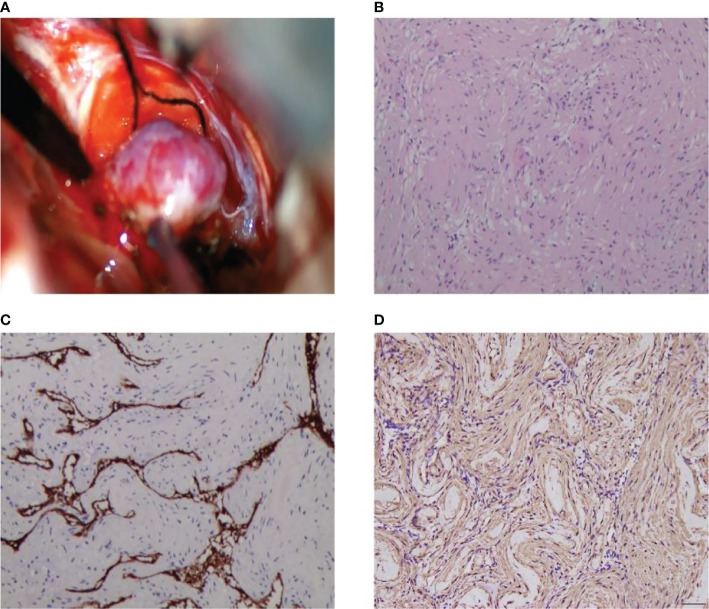
Intraoperative MPC appears as a pink soft-tissue mass **(A)**. Histological and immunohistochemical features of MPC. Hematoxylin-eosin staining showed that the tumor was composed of abundant spindle cells, which grew in concentric circles around small blood vessels **(B)**. Immunohistochemical staining presented CD34 (+) **(C)**, SMA (+) **(D)**. [Original magnifications: **(B–D)** 200×].

**Figure 4:**
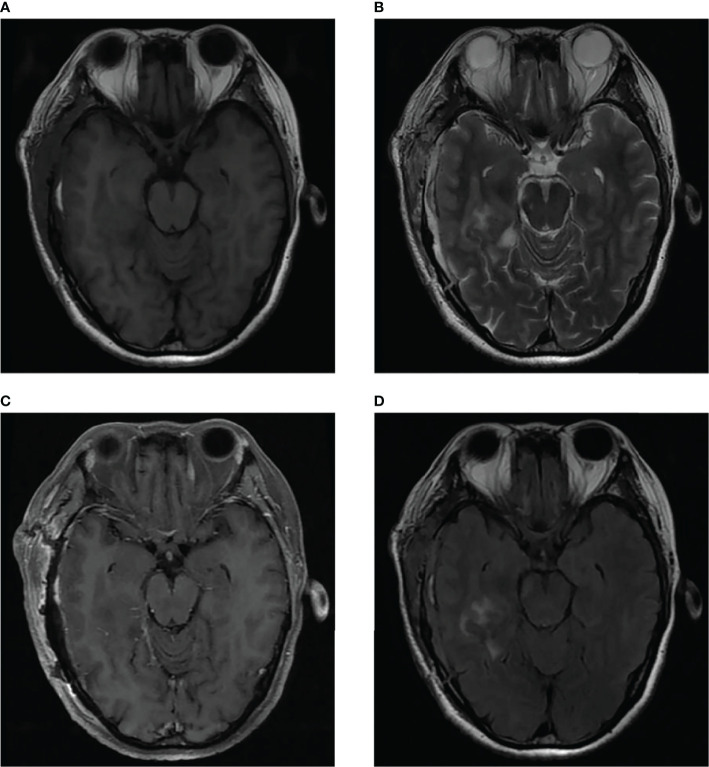
Postoperative brain MRI indicated abnormal long T1-weighted and long T2-weighted signal **(A, B)** in the right part of the pons, which showed high signal on FLAIR imaging **(D)** and no enhancement of the lesion on contrast-enhanced imaging **(C)**.

We present the case of a 45-year-old female with history of suffered dizziness that lasted several seconds two weeks ago. She has no other physical or laboratorial exams alteration; however, brain magnetic resonance imaging (MRI) revealed an aberrant lamellar signal shadow in the pons. The tumor mass was well-defined and measured approximately 1.0 cm×1.2 cm (**Figure 1A**); The signal strength was low in T1-weighted images, while T2-weighted images were higher and more heterogeneous (**Figures 1A, B**). The mass showed a slightly high signal on FlAIR images (**Figure 1D**) and remarkably heterogeneous enhancement on post-contrast enhancement images (**Figure 1C**). Computed tomography angiography (CTA) of the brain detected no marked abnormalities in the intracranial arteries (**Figures 2A, B**). The lesion was in the brainstem and was well defined. MRI indicated low signal intensity on T1-weighted imaging and high signal intensity on T2-weighted imaging. Furthermore, on T1-weighted enhanced imaging, the mass showed a marked nodular enhancement. Based on the above radiological features, we considered it to be a vascular lesion and highly suspected hemangioblastoma. We performed a “Resection of brainstem tumor through right temporal craniotomy” approach to brainstem. Intraoperatively, the tumor was a visualized as a pink mass with a well-demarcated margin and abundant blood supply (**Figure 3A**). The bottom is located lateral to the pons. The excised lesion appeared as a brown soft tissue mass. Histopathological analysis showed considerable number of vasculature-rich spindle cells that developed in concentric circles around tiny capillaries with no evident nuclear division or mitosis, according to histopathological studies (**Figure 3B**). Vimentin (+), CD34 (vasculature+), SMA (+), S100 (-), GFAP (-), D2-40 (-), EBV (-), CK (-), 49EMA (-), Ki67 (1%+) were found in immunohistochemistry (**Figures 3C, D**). This lesion was eventually diagnosed as a MPC based on the histological and immunohistochemical results. After surgery, the patient did not undergo radiotherapy or chemotherapy, and there were no symptoms of recurrence or metastasis. Postoperative brain MRI showed abnormally long T1-weighted and long T2-weighted signals (**Figures 4A, B**) in the right part of the pons; furthermore, we detected a high signal on FLAIR imaging (**Figure 4D**) and no enhancement of the lesion on contrast-enhanced imaging (**Figure 4C**).

## Discussion

Myopericytoma (MPC) was first reported by Granter et al. in 1998 (1). It is a soft tissue tumor that originates from perivascular myoid cells and exhibits features of smooth muscle and vascular bulb cells. It is a perivascular tumor along with myofibroma, hemangiopericytoma, and glomangioma (2–4). MPC often occurs in the skin and subcutaneous soft tissues of the extremities, especially in the lower extremities. However, it has been reported to occur in the head, neck, trunk, and visceral organs in the past (5, 6). MPC of the central nervous system is exceptionally rare. Rousseau et al. originally described three cases of intracranial MPC in 2005 (7). Only nine cases of intracranial MPC have been reported (8), and none have been reported in the pons. MPC can be found at all ages; however, it is most common in middle-aged and older women (9). In 2006, Mentzel et al. studied 54 patients with MPC whose ages ranged from 13 to 87 years (median age, 52 years) (10). MPC is normally asymptomatic, and most cases manifest as slow-growing, painless lumps in superficial tissues. Intracranial MPC, on the other hand, has atypical symptoms, such as headache, nausea, and vomiting (3, 9). In this case, the patient was found to have intracranial MPC due to dizziness.

Although most MPCs are benign, there are some instances of malignant MPC (4). They are generally characterized by active nuclear division, cellular anisotropy, and necrosis (11, 12). Histopathological presentation of MPC usually reveals spindle-shaped or ovoid cells, and they have a proclivity to differentiate into perivascular myoid and pericyte cells. It is characterized by tumor cells growing in a concentric or swirling pattern around blood vessels. MPC is mainly positive for smooth muscle actin (SMA), vimentin, and h-caldesmon; however, it is only marginally positive or negative for desmin, and is generally negative for S100, EMA, CD31, and CD34 (13, 14).

Most studies on MPC are case reports due to the rarity of MPC manifestation, especially in the central nervous system. The imaging performance of MPC is summarized with the following features after studying the relevant literature at home and abroad and combining it with the imaging performance of this case. MPC tumor imaging through CT illustrates well-defined tumor boundaries that are typically isodense or hypodense, though calcification can be detected in a few tumors. On T1-weighted imaging, tumors have a low or slightly low signal, whereas on T2-weighted imaging, they have an inhomogeneous high signal. When the tumor is small, the enhancement is generally uniform or heterogeneous; however, when the tumor is large, necrosis and cystic alterations are common in the center of the lesion, resulting in no enhancement in the center of the tumor and considerable enhancement around it on contrast-enhanced scans (14–16). The appearance of the lesion in this case matched the aforementioned features.

Histopathologically, a MPC is primarily composed of large blood vessels and mucus, both of which are coated with spindle-shaped or oval endothelial cells. It is well known that the histological traits of neoplasms frequently disclose their imaging properties. Consequently, the following tumors are usually included in the differential diagnosis: meningioma, solitary fibrous tumor/hemangiopericytoma, hemangioblastoma, and angioleiomyoma (17). 1) Meningiomas are the most common intracranial and extracerebral tumors. They are generally located on the brain’s convex surface, cerebral fossa, and cerebellar vermis, among other places. On CT, tumors adjacent to the bone may be observed as osteophytes. On T1-weighted imaging, they have a mildly low signal, whereas on T2-weighted imaging, they have a mildly high signal. Several of them show “meningeal tail signs,” and central foci of necrosis or calcification. Contrast-enhanced images usually exhibit marked homogeneous enhancement (18). 2) Solitary fibrous tumor/hemangiopericytoma is an uncommon mesenchymal spindle cell tumor that develops in people over the age of 50 years and shows no distinct sex differences. On T2-weighted images, the signal differs for WHO tumors of different grades. These tumors often have hollow blood vessels inside or on the surface, and necrosis, cystic changes, and surrounding edema are more frequent (19). 3) Hemangioblastomas originate from vascular endothelial cells, which are primarily composed of abundant capillaries and mesenchymal cells. They are most often observed below the cerebellar vermis, primarily in the cerebellum, but sometimes in the brainstem and spinal cord. Hemangioblastoma is best diagnosed using CT scan. Imaging manifestations are classified as cystic, solid, mixed cystic, or solid. Most of these tumors are cystic in nature, with attached nodules, and solid tumors are less common than cystic tumors. Magnetic resonance imaging (MRI) is the “gold standard” for differential diagnosis. On T1-weighted enhancement imaging, the tumor shows uniform nodular enhancement, and its cystic portion shows high signal on T2-weighted imaging and no enhancement on contrast-enhanced imaging (20, 21). 4) Angioleiomyoma are mesenchymal tumors consisting of well-differentiated smooth muscle cells and blood vessels with thick walls. The clinical presentation of the tumor is nonspecific and usually occurs in the extremities. On computed tomography (CT), it is usually moderately dense. On T1-weighted images, it exhibits mostly low or equal signal, and on T2-weighted images, it shows mostly high signal intensity. Furthermore, the most prominent attribute was incremental amplification in contrast-enhanced examinations. On immunohistochemical staining, angioleiomyomas are positive for desmin, CD34, and smooth muscle actin, with the latter two being the most sensitive markers (22). Additionally, arteriovenous malformations, cavernous hemangiomas, and neurinomas should be distinguished from MPC.

Surgical resection is the most effective treatment for MPC. Most patients with MPC have better postoperative recovery and less local recurrence (23). After surgical resection of this tumor, a few recurrences have been reported in the literature, which could be related to inadequate removal of the tumor due to difficulty in complete detachment from neighboring tissues or the formation of new primary lesions (11). Consequently, we are convinced that long-term follow-up after tumor removal is necessary in all patients.

In conclusion, MPC is an uncommon benign soft tissue tumor that most often occurs in the extremities. To our knowledge, MPC of the central nervous system is rare. Moreover, patients with pontine MPC have not been previously reported, suggesting that such cases are extremely rare. Histopathologically, the tumor consists of abundant vascular components, indicating that it has an abundant blood supply. Accordingly, its imaging findings have certain characteristics that can provide a differential diagnosis for benign tumors with plentiful intracranial blood supply. We report this meaningful case, along with a review of the relevant literature, to improve the diagnostic process for intracranial MPC in clinical radiology.

## Data Availability Statement

The original contributions presented in the study are included in the article/**Supplementary Material**. Further inquiries can be directed to the corresponding author.

## Ethics Statement

Written informed consent was obtained from the individual(s) for the publication of any potentially identifiable images or data included in this article.

## Author Contributions

MG and XC, manuscript writing. GZ, manuscript revision. YW, conception and critical review. All authors contributed to the article and approved the submitted version.

## Conflict of Interest

The authors declare that the research was conducted in the absence of any commercial or financial relationships that could be construed as a potential conflict of interest.

## Publisher’s Note

All claims expressed in this article are solely those of the authors and do not necessarily represent those of their affiliated organizations, or those of the publisher, the editors and the reviewers. Any product that may be evaluated in this article, or claim that may be made by its manufacturer, is not guaranteed or endorsed by the publisher.
