# Supplementary figures and images for: Pontine Myopericytoma: Case Report and Literature Review
Source: Front Oncol. 2022 May 16;12:903655. doi: 10.3389/fonc.2022.903655 (PMC9148993; doi:10.3389/fonc.2022.903655)

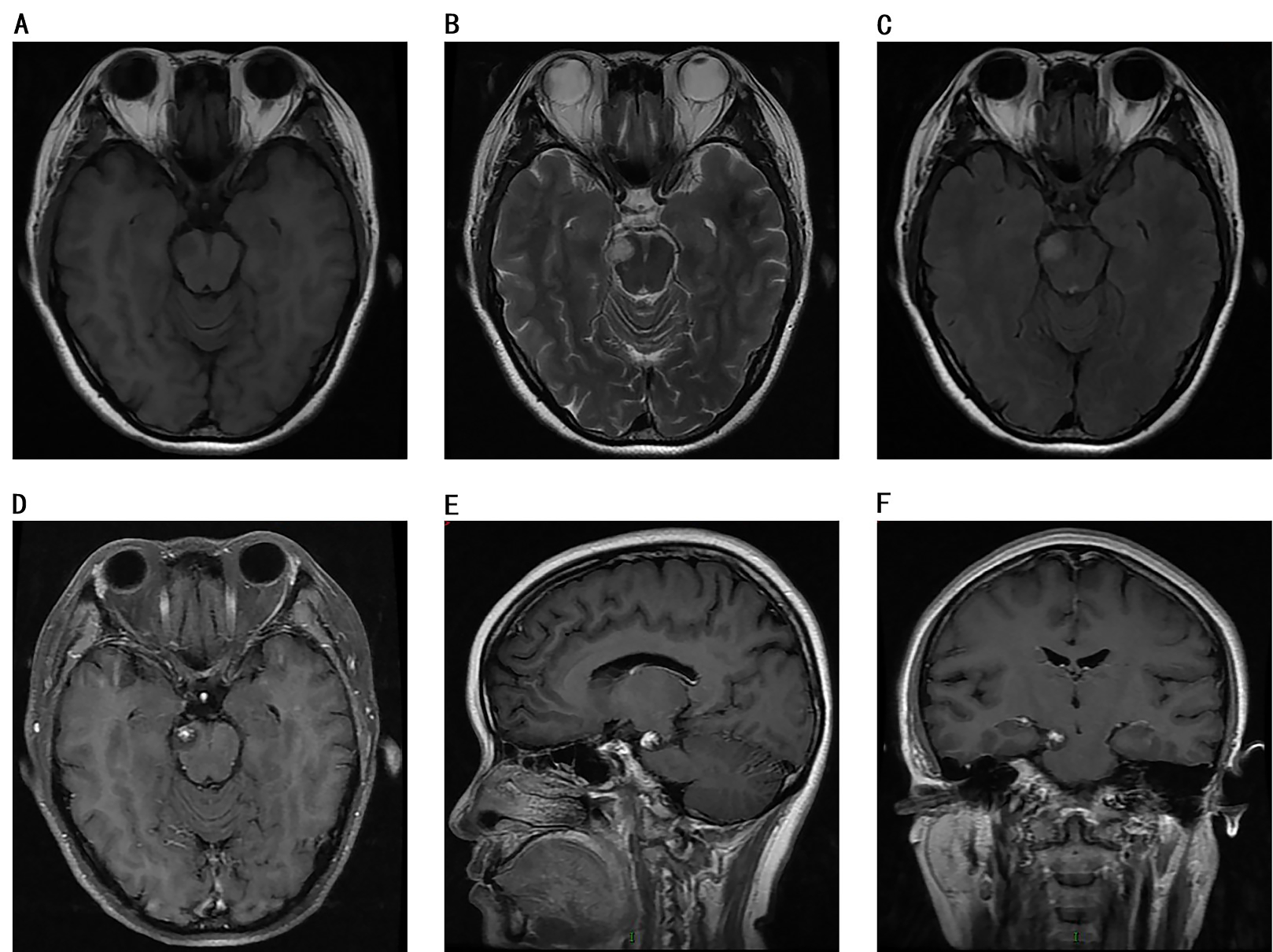

Supplement: Supplementary file 1 [file Presentation_1.zip › Figures/Figure 1.tif]

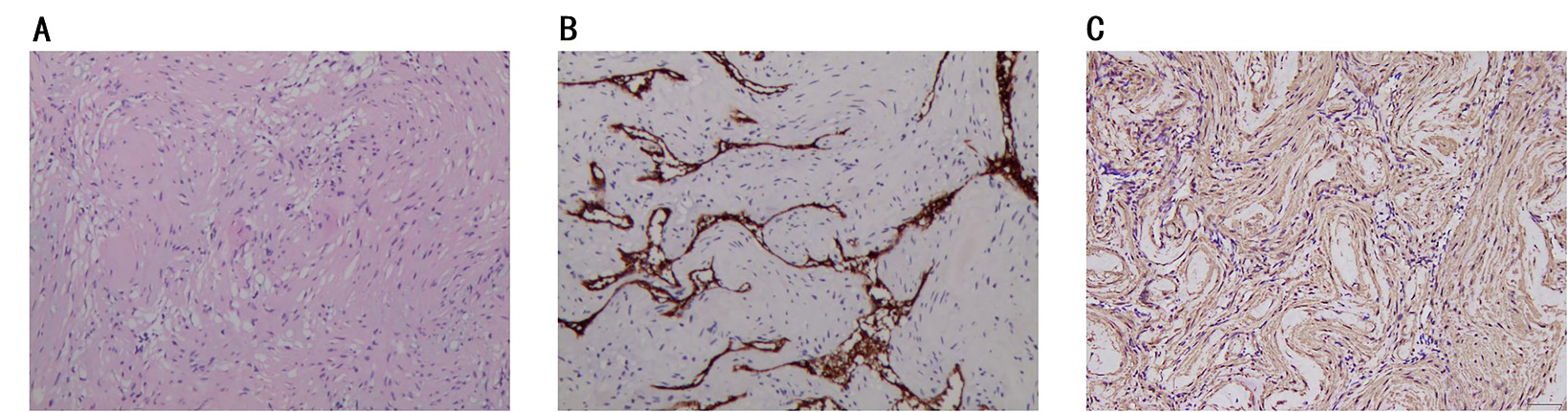

Supplement: Supplementary file 1 [file Presentation_1.zip › Figures/Figure 2.tif]
